# The mediating role of trust in government in intergenerational transmission of fertility intentions

**DOI:** 10.3389/fpubh.2024.1338122

**Published:** 2024-03-01

**Authors:** Jiansong Zheng, Xi Wang, Sujun Xie, Hao Wang, Junxian Shen, Tao Zhang

**Affiliations:** Faculty of Humanities and Social Sciences, Macao Polytechnic University, Macao, Macao SAR, China

**Keywords:** number of siblings, fertility intentions, trust in the local government, education level, China

## Abstract

China's one-child policy was in effect from 1982 to 2015. However, the literature examining the association between people's trust in local government and intergenerational transmission of fertility intentions is scarce. To fill this gap, we investigated the impact of individuals' sibship size on their ideal number of children, the mediating effect of their trust in local government on the issue of fertility between two successive generations, and the moderating effect of education level on sibship size related to trust in local governments. Based on the 2019 Chinese Social Survey data, 2,340 respondents aged 18–35 participated in the analysis. The results showed that (i) individuals' number of siblings significantly positively predicted their ideal number of children; (ii) individuals' number of siblings significantly negatively predicted their trust in the local government, which in turn significantly negatively influenced fertility intentions; (iii) the mediating mechanism was significant in residents with higher levels of education, but not in people with lower degrees of education. Fertility-boosting incentives can prioritize couples who are the only child in their family. It is necessary for local governments to improve their credibility and strengthen their pregnancy-related communication with groups with higher levels of education.

## 1 Introduction

China's one-child policy required a couple to have only one child and lasted from 1982 to 2015 (1, 2). The one-child policy has been in effect for more than 25 years, which is enough time for a generation to give birth to the next one (3). Since the implementation of this policy, the fertility rates in China have been maintained at a lower level. Although from 2021, government policy has allowed couples to have three children, the fertility rate has remained at lower levels (4). The fertility intentions of young people in a country or region can reflect the fertility rate of the society (5). Scholars have proposed many determinants of fertility intentions. For instance, personal factors include income level, education level, and socioeconomic status (6). Family elements include parents' education level, intergenerational parenting, and the number of siblings (7, 8). Social aspects include fertility culture, family policy, and social security reform (6, 9). Accordingly, fertility rates of older generations may reflect social expectations, as well as social norms, and likely affect the fertility intentions of young people (5, 8, 10). Yet, the one-child policy may constitute a break in the family and cultural habits of the population (11).

There is less evidence to support the intergenerational transmission of fertility intentions, especially in China. Though studies find that parents' fertility is positively transmitted to their children's reproductive behaviors, the relevant mechanism remains unclear (6). In other words, individuals with larger sibling numbers are likely to have more children (12). This finding has been validated according to the hypothesis of intergenerational transmission of fertility norms (13). In this hypothesis, the ideal family size is positively correlated across generations and is widely accepted as one of the key mechanisms for the “low fertility trap” in many countries or regions, including China (14). However, on the contrary, single-sibling individuals' experience of childhood loneliness may enhance their willingness to want multiple children (15).

Over three decades, China's one-child policy has dismantled traditional fertility concepts such as “raising children to prepare for old age”—something embedded in the hearts of Chinese nationals (16). Young people who experienced the one-child policy in their childhood likely held the belief that their parents had more siblings, but they were the only child (17). The implementation of the family planning policy by local governments may have been imprinted on the young generation (18). The previous environment of the one-child policy has likely constructed the social norms of low fertility, and this idea was supported by a survey that young people reported lower ideal family size (19). In other words, young generations inherited the fertility attitude of having fewer children with a higher quality of life from their parents (20).

China's three-child policy since June 2021 allows a couple to have three children (21). The implementation of the three-child policy suggests that the previous fertility restriction has now changed to fertility encouragement (9). In this context, the intergenerational transmission of fertility may change (13). Yet, China recorded its first population decline in more than six decades in 2022 (22). Therefore, it is imperative to identify the intergenerational transmission of fertility intentions in contemporary China. To fill this research gap, we focus on the association between sibship size and fertility intentions and its relevant mechanism. Our contribution is discussed below. Evidence from China was used to verify the intergenerational transmission of fertility intentions. In the specific cultural context of China, we explored the mediating role that people's trust in the government played in the intergenerational transmission of fertility. Group differences, especially stemming from the differences in education level, were investigated in the mediating effect.

## 2 Literature review and theoretical hypotheses

Successive Chinese governments have implemented family planning policies over the past four decades (23), resulting in a tortuous battle between the local government and the public over reproductive freedom (24). At the beginning of the one-child policy, the birth propaganda of “Later, Longer, and Fewer” fitted most people's fertility intentions (25). The acceptance of the one-child policy became the basis of people's psychological identity for the implementation of the family policy (26). Although people in the rural areas initially resisted the one-child policy, they eventually accepted the policy after it was adjusted (27). Citizens, in general, were at a disadvantageous position when fighting for their reproductive rights against the government. Yet some of them were able to achieve their fertility goals through many means, including refusing to pay over-birth fees, secretly and illegally bearing children through the back door, and exploiting policy loopholes (1).

The positive intergenerational transmission of fertility intentions may be one of the important reasons for the low fertility of young people in contemporary China (4). Traditional fertility concepts such as “more children, more happiness” have faded away in the minds of most Chinese citizens (28, 29). Most young adults, especially the group growing up during the one-child policy, are the only child in their family and follow the social trend of having fewer children, and they show a lower willingness to have children (13). There is a similar situation across developed countries and regions where government policies to encourage fertility have been met with a lukewarm reception (30). The inertia of the one-child policy over nearly four decades has established the social norm for low fertility (9). The one-child policy norm is practiced by most people of reproductive age and has become a deserved reality (5, 19). People who are the only child in their families face heavier burdens of caring for their parents (31), family-work conflicts (32), and child-raising costs (19, 33) compared to their counterparts with multiple siblings. With the widespread dissemination of information on “abortion” and “contraception” on the Internet, individuals with fewer siblings may form opinions in favor of “having only one child” and “dinky family” (34). Based on this, we developed the following hypothesis:

Hypothesis 1: The number of siblings positively predicts fertility intentions.

People's trust in the local government is an important political resource (35). The level of trust may calibrate the relationship between the local government and the public after their fight for reproductive rights (25). In this context, people with higher levels of trust in the government are easily likely to accept family planning policies (26, 36). Local governments with higher credibility do not have to spend significant resources to persuade people to respond to the family policy (2). Yet, traditional concepts such as “raising children to prepare for old age” would have challenged the implementation of the one-child policy because having more children would have effectively reduced the dependence on the government in the later years of an individual's life (37). When individuals with multiple siblings encounter family emergencies, such as the death of family members due to a possible traffic accident, their social capital is able to generate more buffering capacity for family emergencies, which may result in lower levels of trust in their local government (10, 38, 39). Based on this, we developed the following hypothesis:

Hypothesis 2: The number of siblings individuals have negatively predicts their trust in the local government.

Individuals' trust in the government guarantees them a secure quality of life in their old age (40). This has been proven in countries or regions with higher degrees of welfare systems, such as South Korea, Japan, Hong Kong, Macau, and Singapore (41, 42). For example, South Korea has a complete welfare system with an established series of family-friendly policies and wide coverage benefits to defend women's employment rights, yet the country faces difficulty in raising its fertility rates to the replacement levels (43). With socioeconomic development, China's modern social welfare system is gradually being built, and the social security system is able to care for people in their post-retirement years (44, 45). In the present time, when birth control technology is advanced, less childbearing is physiologically rational (46). Young residents' higher degrees of trust in the local government underlie their confidence in relying on the government in their retirement. Accordingly, youngsters are more likely to marry later and have fewer children, given the time constraints of fast-paced work as well as the long intervals between raising children and relying on them (21, 40). Thus, we developed the following hypothesis:

Hypothesis 3: Individuals' trust in the local government negatively predicts their fertility intentions.
Hypothesis 3a: Individuals' trust in the local government mediates the association between their number of siblings and fertility intentions.

The association between education level and trust in government remains controversial. Scholars have argued that individuals' education level positively predicts their trust in the government for the possible reason that individuals with higher levels of education may have higher cognitive levels and are able to understand the operations of public services (47). However, there is an opposite viewpoint that individuals' education level is negatively related to their trust in governments (48). A plausible explanation is that individuals with higher education levels are more critical of the government's management and service, especially local governance (49). According to social capital theory, siblings can give individuals with higher education levels more social resources to resist social instability, encouraging them to express their critical attitudes against government behaviors (38). Educational level is one of the determinants of socioeconomic status and is also a measure of social capital (50). Individuals with lower degrees of educational attainment may not have strong critical attitudes against government behaviors, even with the support of their siblings. Therefore, people with lower education levels may not readily express lower degrees of trust in government, possibly because they do not have higher degrees of social capital to withstand the risks likely posed by the government (51). Based on this premise, we developed the following hypothesis:

Hypothesis 4: Individuals' education level moderates the correlation between their number of siblings and trust in the local government. Specifically, the higher the sibship size of individuals with higher education levels, the lower their trust in the local government. The sibship size of individuals with lower education levels does not affect their trust in the local government.
Hypothesis 4a: Education level moderates the mediating chain of “number of siblings → trust in the local government → fertility intentions.” Specifically, the higher the sibship size of individuals with high education level, the lower their trust in the local government, which in turn is related to their higher degrees of fertility intention; individuals with lower education level do not have the mediating effect of their trust in the local government in the association between their number of siblings and fertility intentions.

Further, there are demographic information variables predicting fertility intentions. In particular, age may influence decision-making about fertility, and one of the direct reasons is that the optimal reproductive age range of women is 20–40 years (52, 53). The three-child policy may induce men rather than women to show higher fertility intentions because they do not have to experience the physical risks of childbirth, especially in the traditional Chinese culture, such as continuing the bloodline (21). For married individuals, it may be easier to show a preference to have children compared to those in other marital statuses (54). Rural individuals, strongly influenced by traditional concepts of fertility, may exhibit higher fertility intentions compared to urban residents (55). Considering that fertility intention is a psychological variable, subjective socioeconomic status, which measures individuals' subjective feelings regarding their status and likely influences fertility desires, was used as a control variable (56).

**Figure 1:**
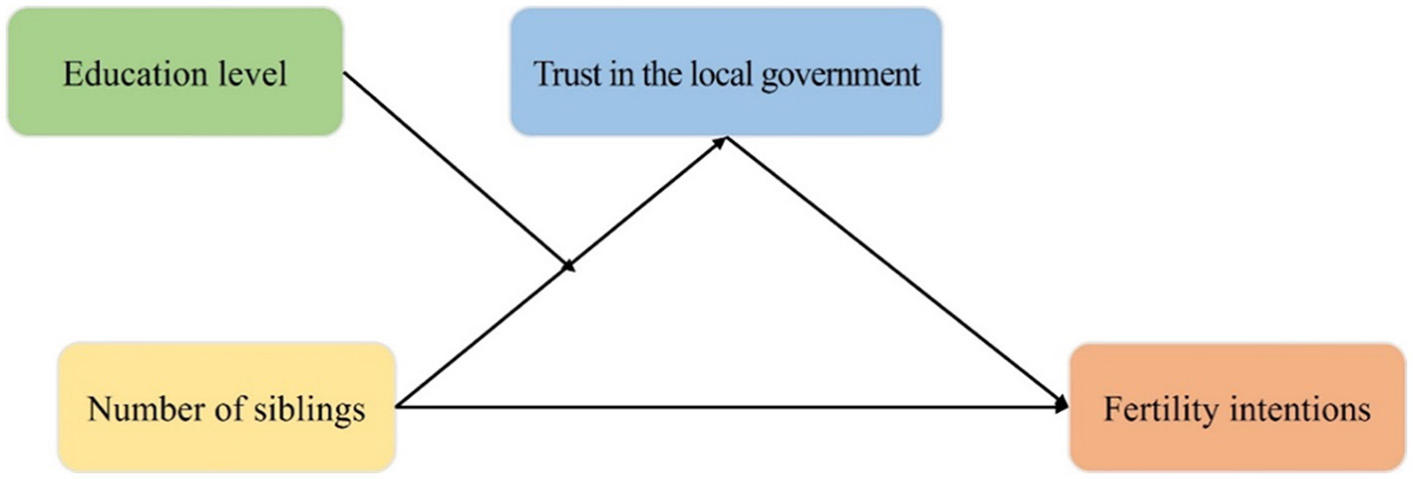
Study framework.

Overall, the positive intergenerational transmission of fertility intentions has been accepted by scholars, but the mechanism in China is still inconclusive (5). In general, individuals' number of siblings is a useful proxy for older generations' fertility rates, and the indicator may reflect the people's responsiveness to family planning policies such as the one-child policy (8). Fertility intentions can be measured using the ideal number of children (36, 57). Local government bodies are policy enforcers during the implementation of family planning policies, and their enforcement of the one-child policy may influence residents' fertility intentions (1). We used data from the 2019 Chinese Social Survey to verify the positive intergenerational transmission of fertility intentions. We also focused on the mediating effect of individuals' trust in the local government on the correlation between the fertility of two successive generations of the same family and the moderating role that education level plays in the effect of the number of siblings on the trust in the local government. The specific research framework is shown in Figure 1.

## 3 Method and variables

The data source was the 2019 Chinese Social Survey (CSS), conducted by the Institute of Sociology, Chinese Academy of Social Sciences. The probability proportional to size (PPS) sampling was used to conduct the survey in 596 villages or committees in 149 cities across 31 provinces in China. The survey population was adults. The questionnaire covered a wide range of indicators, including family, employment, social values, and trust, and can provide empirical support for many disciplines, including sociology, psychology, economics, and public administration. Considering that the target group was born during the implementation of the one-child policy, we chose young Chinese residents aged 18–35 as the research sample. They were born between 1983 and 2000 and are the mainstay of fertility. After missing values of the study variables were removed, 2,340 adults of childbearing age were included in the empirical study.

Fertility intention was the dependent variable, measured by the CSS (2019) item “How many children do you think is the ideal number of children a family usually has?” The number of siblings is the core independent variable, and it was obtained from the CSS (2019) item “Besides you, how many biological siblings do you have from the same parents?”

Trust in the local government was the mediating variable, measured using the CSS (2019) item “Do you trust the township government?” As township governments are important local government bodies that contact people, individuals' trust in township governments can reflect their trust in local governments (48). This item is a 4-point Likert scale, ranging from “1: not at all trust” to “4: very trust.” The higher scores represent greater levels of individuals' trust in the local government.

Education level was the moderating variable, measured by the CSS (2019), with its responses coded as “1: illiteracy, 2: elementary school, 3: middle school, 4: high school, 5: technical secondary school, 6: higher vocational school, 7: junior college, 8: undergraduate, 9: postgraduate.” Education level can be viewed as a continuous variable, with its higher score indicating higher levels of educational attainment of respondents.

Referring to previous studies (5, 8, 9), the control variables included age, gender, marital status, household registration, and subjective socioeconomic status. Age is a continuous variable. Gender is coded as “1: male, 0: female” and marital status as “1: married, 0: other.” Hukou (system of household registration) was coded as “1: rural area; 0: urban area.” Subjective socioeconomic status was measured using the item “What level do you think your current socioeconomic status is in the local area?” This question is a 5-point Likert scale, with higher scores representing higher levels of the individuals' subjective socioeconomic status.

Table 1 shows the descriptive statistics of the study variables. Considering that the ideal number of children is a continuous variable, the linear probability model was used to identify the intergenerational transmission of fertility. Individuals' trust in local government likely mediates the association between their number of siblings and fertility intentions, and their education level may moderate the mediation mechanism by moderating the effect of sibship size on trust in the local government. We employed the mediation analysis method, the Sobel mediation test, the simple slope test, and the Bootstrap method to verify the moderated mediating mechanism (58, 59).

**Table 1:** Descriptive statistics.

**Variables**	**Mean**	**SD**	**Percentages**
Ideal number of children	1.936	0.543	
Number of siblings	0.623	0.730	
Trust in the local government	2.819	0.810	
Education level	5.331	2.274	
Age	26.92	5.293	
Gender (1 = male)			39.97
Marital status (1 = married)			58.67
Hukou (1 = rural area)			69.04
Subjective socioeconomic status	2.365	0.881	

N = 2,340.

We reported the mean and standard deviation of the continuous variables, including the ideal number of children, number of siblings, trust in the local government, education level, age, and subjective socioeconomic status.

The corresponding percentages of categorical variables as 1 involving gender, marital status, and hukou are displayed.

SD, standard deviation.

## 4 Results

### 4.1 The effect of the number of siblings on fertility intentions

Table 2 shows the regression results regarding the intergenerational transmission of fertility. There were only dependent and independent variables in Model 1. Based on model 1, model 2 was added with controls including age, gender, marital status, hukou, and subjective socioeconomic status. Based on model 2, model 3 was added with trust in the local government. Model 4 was added with education level based on model 3. The results indicated that participants' number of siblings significantly positively predicted their fertility intentions and the predictive relation remained robust in all four models. Individuals with higher numbers of siblings tended to have more children. Hypothesis 1 was verified.

**Table 2:** Linear probability model results with the ideal number of children as the independent variable.

	**Model 1**	**Model 2**	**Model 3**	**Model 4**
Number of siblings	0.136^***^	0.138^***^	0.136^***^	0.129^***^
	(0.016)	(0.017)	(0.017)	(0.017)
Age		−0.002	−0.002	−0.003
		(0.003)	(0.003)	(0.003)
Gender		0.074^**^	0.073^**^	0.069^**^
		(0.024)	(0.024)	(0.024)
Marital status		0.036	0.035	0.017
		(0.030)	(0.030)	(0.031)
Hukou		0.071^**^	0.065^**^	0.035
		(0.024)	(0.024)	(0.027)
Subjective socioeconomic status		0.011	0.014	0.017
		(0.013)	(0.013)	(0.013)
Trust in the local government			−0.030^*^	−0.028^*^
			(0.014)	(0.014)
Education level				−0.017^**^
				(0.006)
Constants	1.849^***^	1.772^***^	1.862^***^	2.000^***^
	(0.015)	(0.075)	(0.085)	(0.098)
*N*	2,340	2,340	2,340	2,340
*R* ^2^	0.032	0.040	0.042	0.045
Adjusted *R*^2^	0.0316	0.0377	0.0392	0.0422
*F*	77.34^***^	16.25^***^	14.64^***^	13.88^***^

Standard errors were reported in parentheses.

^***^p < 0.001.

^**^p < 0.01.

^*^p < 0.05.

Models 2, 3, and 4 displayed the effects of control variables on fertility intentions. Age was not a significant predictor for the ideal number of children. There was a significant gender difference in fertility intentions, and the results remained robust across the models; men specifically exhibited a higher ideal number of children than women. Marital status did not play a significant predictive role in the ideal number of children. Residents from rural areas rather than urban areas reported a higher ideal number of children, and this effect disappeared when education level was added to the model. No empirical evidence supported the significant effect of subjective socioeconomic status on the ideal number of children. Trust in the township government significantly negatively predicted the ideal number of children. Higher levels of education of an individual were related to the lower ideal number of children.

### 4.2 Trust in the local government as a mediator

Table 3 shows the regression results with individuals' trust in local government as a mediating variable. The results of Models 9 and 11 showed that individuals' number of siblings significantly negatively predicted their trust in the local government. Hypothesis 2 was verified. Results of Models 10 and 12 revealed that individuals' trust in the local government significantly negatively predicted their ideal number of children, and Hypothesis 3 was verified. Combining the results of Models 1 and 2 in Table 2, we can draw a conclusion that individuals' trust in the local government plays a partially mediating role in the association between their number of siblings and fertility intentions. In other words, individuals' number of siblings significantly negatively predicted their trust in the local government, which in turn significantly negatively predicted their fertility intentions. The evidence was in favor of Hypothesis 3a. To further examine the robustness of this mediating effect, the Sobel test was utilized to verify the significance of the mediating mechanism. The *Z*-statistic of the Sobel test for the mediating effect was 2.198 (*p* = 0.028) for the model without control variables, and Hypothesis 3a was again verified. However, when control variables were added, this mediation effect was marginally significant (*Z* = 1.691, *p* = 0.091). The mediating role that individuals' trust in the local government plays in the correlation between their number of siblings and the ideal number of children may also be influenced by other factors, such as education level.

**Table 3:** Linear probability model results for mediating analysis.

**Dependent variable**	**Model 9**	**Model 10**	**Model 11**	**Model 12**
	**Trust in the local government**	**Ideal number of children**	**Trust in the local government**	**Ideal number of children**
Number of siblings	−0.104^***^	0.133^***^	−0.066^**^	0.136^***^
	(0.024)	(0.016)	(0.025)	(0.017)
Trust in the local government		−0.034^*^		−0.030^*^
		(0.014)		(0.014)
*Z*	2.198^*^		1.691^†^	
Control variables	No	No	Yes	Yes
*N*	2,340	2,340	2,340	2,340
*R* ^2^	0.008	0.035	0.037	0.042
Adjusted *R*^2^	0.00782	0.0339	0.0347	0.0392
*F*	19.44^***^	41.98^***^	15.01^***^	14.64^***^

Standard errors were reported in parentheses.

^***^p < 0.001.

^**^p < 0.01.

^*^p < 0.05.

^†^p < 0.1.

### 4.3 Education level as a moderator

**Figure 2:**
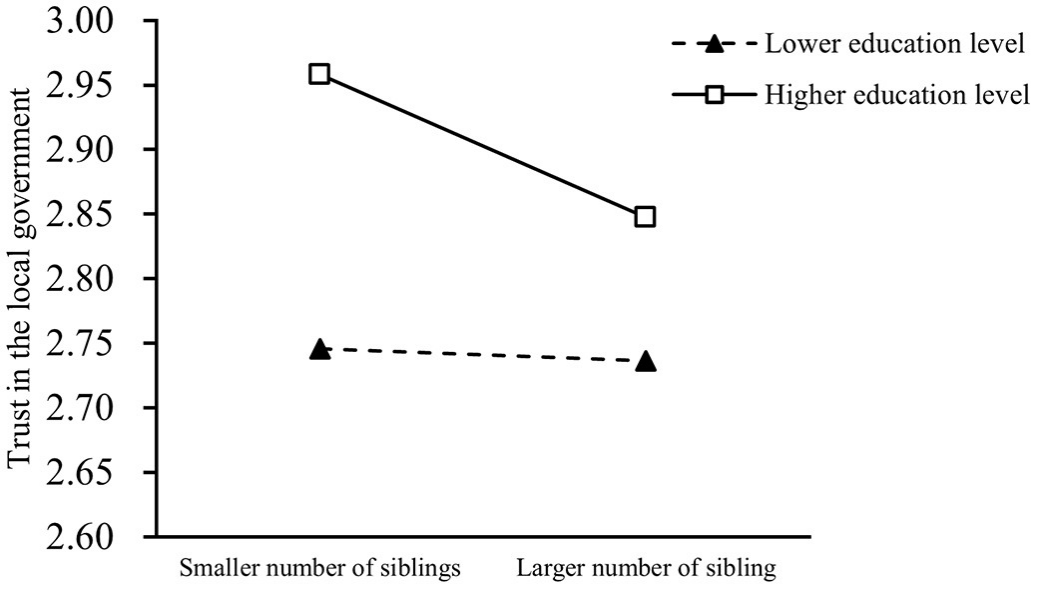
The results of the simple slope test.

Table 4 shows the effect of the interaction term between individuals' number of siblings and education level on their trust in the local government. The results indicated that the interaction term significantly predicted trust in the local government for the model without controls (*p* = 0.037 in Model 13, *p* = 0.008 in Model 14, and *p* = 0.068 in Model 15), and Hypothesis 4 was verified. When control variables were included, the interaction term did not significantly predict trust in the local government (*p* = 0.081 in Model 16, *p* = 0.435 in Model 17, and *p* = 0.134 in Model 18). To clarify how the interaction term affects trust in the local government, we utilized a simple slope test to examine how individuals' number of siblings with different levels of education affects their trust in the local government (Figure 2). In this case, individuals' smaller and larger numbers of siblings were measured by the samples' mean minus and plus one standard deviation, respectively. The mean minus and plus one standard deviation of individuals' education levels were calculated to characterize the lower and higher education levels of the participants, respectively. The simple slope results indicated that in terms of individuals with higher education levels, their number of siblings significantly negatively predicted their trust in the local government (*Effect* = −0.111, *p* = 0.009), while the number of siblings of individuals with lower education levels had no significant effect on their trust in the local government (*Effect* = −0.009, *p* = 0.750).

**Table 4:** Linear probability model results for moderating analysis with trust in the local government as the independent variable.

	**Model 13**	**Model 14**	**Model 15**	**Model 16**	**Model 17**	**Model 18**
Interaction item	−0.010^*^	0.021^**^	−0.019^†^	−0.008^†^	0.006	−0.016
	(0.005)	(0.008)	(0.010)	(0.005)	(0.008)	(0.010)
Number of siblings		−0.194^***^	0.027		−0.095^*^	0.018
		(0.041)	(0.055)		(0.044)	(0.055)
Education level			0.060^***^			0.039^***^
			(0.010)			(0.011)
Control variables	No	No	No	Yes	Yes	Yes
Constants	2.846^***^	2.877^***^	2.537^***^	2.991^***^	2.964^***^	2.676^***^
	(0.021)	(0.022)	(0.060)	(0.113)	(0.114)	(0.140)
*N*	2,340	2,340	2,340	2,340	2,340	2,340
*R* ^2^	0.002	0.011	0.027	0.036	0.037	0.043
Adjusted *R*^2^	0.001	0.010	0.026	0.033	0.035	0.039
*F*	4.37^***^	13.30^***^	21.49^***^	14.33^***^	12.95^***^	12.96^***^

Standard errors were reported in parentheses.

^***^p < 0.001.

^**^p < 0.01.

^*^p < 0.05.

^†^p < 0.1.

### 4.4 Moderated mediation model results

Combining the results of Models 4, 12, and 18^1^, the SPSS and Process 4.2 were used to construct a moderated mediation model with fertility intentions as the dependent variable, the number of siblings as the independent variable, trust in the local government as the mediating variable, and education level as the moderating variable (Figure 1). The Bootstrap method based on 5,000 random samples was utilized to test this moderated mediation model (Table 5). The results showed that, for individuals with higher education levels, their trust in the local government significantly mediated the relationship between their number of siblings and fertility intentions (95% Bootstrap confidence interval excluded 0). In terms of individuals with lower levels of education, the mediating effect was not significant (95% Bootstrap confidence interval included 0). Hypothesis 4a was supported.

**Table 5:** The results of the moderated mediation model.

**Education level**	**Effect value**	**Bootstrap SD**	**95% Bootstrap LLCI**	**95% Bootstrap ULCI**
*M* – SD	0.0003	0.0012	−0.0019	0.0030
*M* + SD	0.0035	0.0022	0.0010	0.0088

SD, standard deviation; M, mean; LLCI, lower limit of confidence interval; ULCI, higher limit of confidence interval.

### 4.5 Robustness check

Table 6 shows the results of the robustness test using linear probability models. The aim of the robustness test is to avoid possible biased estimation due to the selection of age intervals. Therefore, we chose individuals in different age intervals to empirically test a positive correlation between the fertility of successive generations in the same family. Models were conducted using samples of individuals aged 18–50, 20–35, and 20–40 years old, representing a wider, narrower, and cross-age range than the original model. Model results from the samples of different age groups indicated a significant positive intergenerational transmission of fertility. Model robustness and sample representativeness were ensured.

**Table 6:** The results of the robustness check.

	**Dependent variable: ideal number of children**					
	**18–50 years old**		**20–35 years old**		**20–40 years old**	
	**Model 19**	**Model 20**	**Model 21**	**Model 22**	**Model 23**	**Model 24**
Number of siblings	0.078^***^	0.063^***^	0.134^***^	0.131^***^	0.116^***^	0.104^***^
	(0.007)	(0.008)	(0.016)	(0.017)	(0.012)	(0.013)
Controls	No	Yes	No	Yes	No	Yes
*N*	5,175	5,175	2,088	2,088	2,869	2,869
*R* ^2^	0.021	0.030	0.033	0.045	0.030	0.040
Adjusted *R*^2^	0.021	0.029	0.032	0.042	0.030	0.038
*F*	109.40^***^	27.08^***^	70.90^***^	16.32^***^	88.36^***^	19.75^***^

^***^p < 0.001.

## 5 Discussion

This study validated the positive intergenerational transmission of fertility intentions, which may be China's evidence for a family of origin in line with fertility intentions. The Chinese one-child policy controlled the population explosion and the majority of citizens adhered to this policy (25). Yet, some competed with the government for their reproductive freedom, using a range of tactics, including illegal birth, over-birth, and exploiting policy loopholes to achieve the goal of having more children (1, 26).

Individuals with parents who responded positively to the one-child policy exhibited higher levels of trust in the local government and lower degrees of fertility intentions. These results were robust after adding control variables. People directly contacted local government bodies regarding the latter's conduct of family policies, and people's trust in the government may correlate with their response to the implementation of family policies (32, 60). This indicates that local governments' actions are related to residents' intentions, such as fertility desires (1).

In our study, empirical evidence supports the negative association between individuals' education level and their fertility intentions. Individuals' education level is positively associated with their socioeconomic status and likely correlates with a diversity of values. Therefore, people with higher degrees of education attainment are more receptive to ideas regarding DINK or fewer children (27, 61). Moreover, individuals with higher levels of educational attainment have higher opportunity costs for raising children, which may lead them to prefer having fewer children (55). Groups with higher education levels also exhibit higher levels of trust in the local government, which is consistent with a few previous studies (47, 62). However, we found that individuals with higher education levels and a higher number of siblings exhibited a lesser extent of trust in the local government, which in turn negatively predicted their ideal number of children. A possible reason is that individuals with higher education levels have higher degrees of cognition and can better understand government behaviors. Consequently, they may also hold critical attitudes when processing family planning policies (48, 62). These critical attitudes are consolidated when individuals with higher education are backed by their siblings, who give them social capital. As a result, it is easier for them to show lower levels of trust in the local government (38, 47). In this case, for individuals with higher education levels, there is a mediating effect of trust in their local government in the association between their number of siblings and the ideal number of children. Groups with lower levels of education are mainly engaged in manual work and are more likely to be submissive to the local government, and therefore, they may not generally have critical mindsets toward government actions (47).

We studied the effects of control variables on fertility intentions. The male sub-sample, relative to the female sub-sample, had higher ideal numbers of children, possibly because women need to commit more in terms of their body, economy, and time when considering fertility decisions (19, 21). Compared to urban residents, rural individuals showed higher fertility intentions, probably because the traditional fertility concepts such as “more children, more happiness” are still prevalent in rural areas rather than urban regions (63). We did not find any significant predictive effects of age, marital status, and subjective socioeconomic status on fertility intentions, possibly because informational developments homogenized differences in age, marriage, and socioeconomic status of fertility intentions (21, 64).

With the implementation of the three-child policy, local governments' focus has shifted from restricting fertility to encouraging it (5). While engaged in fertility-related work, the government needs to pay attention to the positive intergenerational transmission of fertility intentions (8, 13). The government can offer policy advantages to individuals without siblings, such as higher cash subsidies for childbirth, more flexible work arrangements, and longer maternity leave periods (8, 57). Additionally, the government should focus on groups with higher education levels, who have a significant mediating effect on their trust in the local government in the intergenerational transmission of fertility intentions. Local governments should offer services related to childbirth, especially for people with higher education levels (65, 66).

There are still some shortcomings in this study. Firstly, the cross-sectional data that we used failed to investigate the causality of intergenerational transmission of fertility intentions. Secondly, fertility intention is a psychological variable influenced by a larger number of unobservable determinants (67). This may lead to a lower level of *R*^2^ as well as adjusted *R*^2^ in the models; however, the accuracy of the models was not affected (68).

Although we identified the mediation effect of trust in local government in the intergenerational transmission of fertility, the gender imbalance caused by the one-child policy was not empirically verified. A negative effect of China's one-child policy on the population is an imbalance in the ratio of men to women, where the number of male newborns is statistically significantly greater than the number of female newborns during this period (29, 69, 70). When considering the reproductive behaviors of the successive generation, a decrease in the number of women born during the one-child policy led directly to a reduction in the number of newborns (71). Yet, this study used the data of the family or individual level to verify the mechanisms between the fertility of two successive generations in the same family, and people's genetic components and fertility attitudes were found to be the main mediators between the fertility of successive generations (20). Although economic development in a country or region experiences a decrease in fertility (72, 73), the intergenerational transmission of fertility may be more dependent on cultural, psychological, and biological determinants (74). In this case, we found that China's evidence in favor of the intergenerational transmission of fertility is mediated by psychological factors such as people's trust in the government.

## 6 Conclusion

China's one-child policy and intergenerational transmission of fertility intentions may be a factor in low fertility rates in contemporary China. To verify the intergenerational transmission of fertility intentions and its mechanism in China, we conducted linear probability models and mediation as well as moderation methods by using a sample among 2,340 adults of reproductive age from the 2019 Chinese Social Survey. The results showed that individuals' number of siblings was significantly positively associated with their ideal number of children. Education level significantly moderated the mediating effect of trust in the local government in the association between the number of siblings and fertility intentions. Highly educated individuals with a higher sibship size were significantly negatively related to their trust in the local government, which in turn negatively impacted their fertility intentions; this mediating effect was not significant for individuals with lower education levels.

## Data availability statement

Publicly available datasets were analyzed in this study. This data can be found at: csqr.cass.cn.

## Ethics statement

Ethical approval was not required for the study involving humans in accordance with the local legislation and institutional requirements. The studies were conducted in accordance with the local legislation and institutional requirements. Written informed consent to participate in this study was not required from the participants in accordance with the national legislation and the institutional requirements.

## Author contributions

JZ: Conceptualization, Methodology, Writing – original draft, Writing – review & editing, Data curation, Formal analysis, Funding acquisition, Investigation, Software, Validation. XW: Supervision, Writing – review & editing, Conceptualization. SX: Writing – review & editing, Formal analysis, Investigation. HW: Data curation, Methodology, Writing – review & editing. JS: Writing – review & editing, Data curation, Visualization. TZ: Funding acquisition, Supervision, Writing – original draft, Writing – review & editing, Conceptualization, Formal analysis, Project administration, Validation.

## Footnotes

^1^Combining Models 1, 10, and 15, the moderated mediation model without control variables was constructed. This Model's results, especially the significance of the key variables, remained consistent with the results of the mediated mediation model with the control variables, so they were not repeated. Readers can request results from the corresponding author.
